# Increased risks of Maternal Mental Health Conditions Following the COVID-19 Pandemic

**DOI:** 10.1089/whr.2024.0070

**Published:** 2024-10-21

**Authors:** Anita Zhou, Allison Zetterman, Megan Ott, Colman Freel, Kayla Adams, Andrea Rodriguez-Dasta, Rebekah Rapoza, Rebecca Drakowski, Sarah Sweeney, Alyssa Freeman, Matthew VanOrmer, Melissa Thoene, Elizabeth Lyden, Charmayne R. Adams, Gurudutt Pendyala, Corrine Hanson, Ashley J. Blount, Ann Anderson-Berry

**Affiliations:** ^1^ Department of Pediatrics, University of Nebraska Medical Center, Omaha, Nebraska, USA.; ^2^ College of Arts and Sciences, University of Nebraska at Lincoln, Lincoln, Nebraska, USA.; ^3^ College of Medicine, University of Puerto Rico at Mayagüez, Mayagüez, Puerto Rico.; ^4^ College of Public Health, University of Nebraska Medical Center, Omaha, Nebraska, USA.; ^5^ Student Health, Counselling, and Wellness and Student Affairs & Counselor Education, Gonzaga University, Spokane, Washington, USA.; ^6^ Department of Anesthesiology, University of Nebraska Medical Center, Omaha, Nebraska, USA.; ^7^ College of Allied Health Professions, University of Nebraska Medical Center, Omaha, Nebraska, USA.; ^8^ Department of Counselling, University of Nebraska at Omaha, Omaha, Nebraska, USA.

**Keywords:** anxiety, COVID-19, depression, mental health, pregnancy

## Abstract

**Background:**

Women are at an increased risk of developing psychiatric conditions such as anxiety and depression during pregnancy. Psychiatric conditions during pregnancy can put mothers and fetuses at risk of worse physical and mental health before, during, and after the completion of a pregnancy. Previous research indicates that women pregnant during COVID-19 were at a greater risk of developing mental health conditions and being exposed to stressors. While most of the research in the field focuses solely on maternal mental health or interactions between stressors and maternal mental health, little research has been done comparing maternal mental health, demographic variables, and socioeconomic variables across pre-, during-, and post-COVID-19 time periods.

**Methods:**

We conducted *an observational cohort study* of 703 mothers divided into pre-, during-, and post-COVID-19 groups based on time of enrollment.

**Results:**

Rates of maternal anxiety (*p* < 0.001), medication use for anxiety (*p* < 0.001), depression (*p* < 0.001), medication use for depression (*p* < 0.001), history of postpartum depression (*p* < 0.001), and medication use for other psychiatric conditions (*p* < 0.001) significantly increased during COVID-19 and remained at elevated levels post-COVID-19 compared with pre-COVID-19. Income >150% of the poverty level (*p* = 0.003), food security level (*p* = 0.010), marital status (*p* = 0.001), and private insurance type (*p* < 0.001) were significantly increased during COVID-19 compared with pre-COVID-19 levels.

**Conclusions:**

Future work should focus on stratifying the effects of the COVID-19 pandemic on maternal mental health by race and ethnicity and establishing guidelines to support maternal mental health during epidemics and pandemics.

## Introduction

On March 11, 2020, the World Health Organization declared a global COVID-19 pandemic caused by severe acute respiratory syndrome coronavirus 2 (SARS-CoV-2).^1^ The COVID-19 pandemic began an extended period of societal lockdowns, which increased unemployment and resulted in acute social isolation for many individuals—particularly those infected with SARS-CoV-2.^1^ While mental health worsened for all groups, women saw a 44%–75% higher increase in poor mental health outcomes compared with men.^2,3^ Variability in mental health is often attributed to major life changes, including new work-from-home policies and employment loss, events that may contribute to increased alcohol/drug use and suicide rates.^2,3^ Considering the effects of COVID-19 on mental health within the general population, it is likely that pregnant women suffered similar increases in the risks for mental health and substance use disorders.

Pregnancy is a period of rapid and dramatic physiological change, during which the risk and consequences of mental health issues are magnified. Pregnant women are at increased risk of developing psychiatric conditions compared with nonpregnant women, which can persist beyond the conclusion of pregnancy.^4,5^ Symptoms of anxiety or depression are reported in 30%–58% of pregnancies, with lower rates of phobias, mania, and obsessive-compulsive disorders.^4,5^

Anxiety is one of the most common psychiatric conditions in pregnancy. Up to 25% of women develop diagnoses of anxiety during gestation, with an additional 20% developing anxiety in the postpartum period.^5,6^ Anxiety can arise due to maternal worries, including concerns about the timing of labor, prior negative experiences in the healthcare system, and newborn health.^5^ Anxiety in pregnancy is often associated with fatigue, biological changes due to pregnancy, and mood changes that may also include depressive symptoms.^7^ Depression is another common psychiatric condition, with 10%–40% of women developing depression during gestation.^5,8^ Pregnant women who develop anxiety or depression may be at greater risk of giving birth preterm, undergoing cesarean section (C-section), and developing postpartum depression (PPD).^6,9,10^ PPD is characterized by depressive symptoms along with symptoms of anxiety and increased arousal and lability of mood.^9,10^ This condition affects up to 19% of mothers between 2 weeks and 1.5 years after delivery.^9,10^ Furthermore, PPD is often comorbid with other postpartum mental health conditions, including anxiety disorders, and is associated with an increased risk of obsessive-compulsive disorder or thoughts of harming one’s infant.^5,11^

Current research indicates that pregnant women who develop mental health conditions are at risk of developing worse pregnancy outcomes. For pregnant women, mental health conditions may increase the risk for adverse outcomes, including hypertension over the course of pregnancy and complications during delivery, as well as increased risk of alcohol use, recreational substance use, and smoking as coping mechanisms.^5,12^ In infants, poor maternal mental health may contribute to outcomes such as preterm birth, low birth weight, and altered development of cognition and language skills.^13^ As such, mental health conditions in pregnant women not only affect the health outcomes of pregnant women themselves but also have a transgenerational impact on their infants.

Recent investigations have revealed an increased prevalence of anxiety and depression in pregnant women following the onset of the COVID-19 pandemic as compared with pre-pandemic levels.^14^ During this period, pregnant women frequently indicated health care concerns, such as trouble accessing hospital services, limited support from partners and family during delivery, fear of transmitting COVID-19 to their offspring *in utero* or at delivery, and worry that maternal COVID-19 infection might impart developmental defects or delays to offspring.^14^ These pandemic-era stressors may have levied a heavy toll on maternal mental health, affecting the broader health of both mother and child.

Previous research has compared the mental health of pregnant women before and following the onset of the COVID-19 pandemic; however, less work has been done comparing maternal mental health, medication use, substance use, and socioeconomic variables across these time periods. Our study aims to examine maternal mental health in pregnancy along with medication use, substance use, and socioeconomic variables to further describe and clarify the effect of the COVID-19 pandemic on maternal mental health in an observational cohort of pregnant women in the midwestern United States.

## Materials and Methods

An institutional review board (IRB)-approved study (#112-15-EP) enrolled women presenting for delivery at the University of Nebraska Medical Center between June 2015 and August 2023 into an observational cohort study. Infants considered wards of the state and infants with several congenital anomalies were excluded. Additionally, women with metabolism-altering medical conditions, such as inborn errors of metabolism, liver disease, or kidney disease, were excluded from recruitment into the study. Due to hospital safety policies, women with a positive COVID-19 test during their hospital admission were also excluded from enrollment in this study. Mothers were divided into the following three groups based on their delivery dates: pre-, during-, and post-COVID-19. The pre-COVID-19 group was defined as delivery between 06/01/15 and 02/29/20, the during-COVID-19 group was defined as delivery between 03/01/20 and 12/31/21, and the post-COVID-19 group was defined as delivery between 01/01/22 and 08/31/23. We chose 12/31/21 as the cutoff between during- and post-COVID-19 groups as vaccinations were widely available to people by the end of 2021 and societal lockdown restrictions, such as mask mandates or remote school/work requirements, were beginning to lift throughout the state of Nebraska.^15^ Additionally, as unemployment returned to near pre-pandemic levels,^16^ multiple federal programs to address the economic impact of COVID-19 expired by December 2021, including stimulus payments, expanded unemployment, and expanded child tax credits.^17–19^ It is important to note that women in the post-COVID-19 group were still impacted by the COVID-19 pandemic; however, the movement to return to “normalcy” throughout 2022 and 2023 may have blunted the impact of the pandemic on maternal mental health. This research fills a gap in the scientific literature by elucidating the acute impact of the COVID-19 pandemic on maternal mental health (during-COVID-19 group) as well as the chronic impact (post-COVID-19 group).

Maternal mental health history was documented *via* review of the electronic health record (EHR) as described below.

### Primary outcome measures

#### Anxiety, depression, and other mental health conditions

Anxiety, depression, and other mental health conditions were determined from clinical diagnoses documented in the EHR during the first postpartum visit. This included new diagnoses during the first postpartum visit, as well as previously diagnosed ongoing anxiety, depression, or other mental health conditions.

#### History of anxiety, history of depression, and history of PPD

Any prior history of anxiety, history of depression, and history of PPD was determined from the past medical history of the patient located within the EHR.

#### Psychiatric medication use

Psychiatric medication use was determined from the medications prescribed to the patient after the first postpartum visit.

#### Illicit substance use and alcohol use

Illicit substance use and alcohol use were determined from the past social history component of the EHR at the first postpartum visit. As substance use was self-reported by participants to their physician prior to documentation in the EHR, the accuracy of this outcome may be limited by social desirability bias.

#### Smoking status

Smoking status was determined from the study’s smoking and vaping questionnaire given to mothers, which focuses on variables such as type of smoking and smoking status. This outcome may be limited by social desirability bias.

Other clinical variables collected from the EHR included infant birthweight, corrected gestational age (CGA) at birth, delivery mode, sex of the infant, insurance type, COVID-19 vaccination history, and incidence of COVID-19 infection during pregnancy. Socioeconomic variables were collected using study questionnaires and included education level, marital status, renting or owning a home, transportation security, employment status, income, self-reported barriers to accessing healthy foods, and enrollment in the Supplemental Nutritional Assistance Program (SNAP) or Women Infants and Children (WIC). Income to poverty level was calculated using the U.S. Department of Health and Human Services’ Federal Poverty Guidelines.^20^ Food security was assessed using the U.S. Department of Agriculture’s Household Food Security Module.^21^

Descriptive statistics were generated for all variables, including means and standard deviations (SDs) for continuous variables and frequencies and percentages for categorical variables. Chi-squared analyses were done to compare outcome variables with pandemic time periods. For continuous variables, analyses of variance (ANOVAs) were completed. Tukey’s method was used to adjust for multiple comparisons for both chi-squared and ANOVA tests. Participants were excluded from analyses for which they were missing data but included in all other analyses. Analysis was performed using SAS Version 9.4. A *p*-value <0.05 was considered statistically significant.

## Results

### Participant demographics

A total of 709 mothers were included in this observational cohort study. In our analysis, 525 mothers (74.0%), 107 mothers (15.1%), and 77 mothers (10.9%) fell into pre-, during-, and post-COVID-19 groups, respectively. The mean maternal age was 29 years, with a SD of 5.5 years. Of these women, 458 (63.4%) were non-Hispanic White, 102 (15.6%) were non-Hispanic Black or African American, 59 (8.8%) were Hispanic, and the remaining 73 women (12.2%) identified as other races/ethnicities or declined to provide their race/ethnicity. The mean CGA at birth was 38.3 weeks with a SD of 2.8 weeks (*n* = 691). For delivery mode, 520 (73.4%) women delivered vaginally, while 188 (26.6%) delivered via C-section. Women in the post-COVID group were significantly more likely to deliver vaginally compared with women in the pre-COVID (*p* < 0.001) and during-COVID groups (*p* < 0.001). All demographic variables assessed in this study are in Table 1.

**Table 1.** Descriptive Variables (Categorical and Continuous) for Study Population

Variable	Total study population *n* mean (SD)	Pre-COVID *n* mean (SD)	During-COVID *n* mean (SD)	Post-COVID *n* mean (SD)	*p*-Value
Birthweight	708	525	106	77	0.316
	3195.79 (692.50)	3190.50 (715.25)	3146.42 (658.73)	3299.83 (565.60)	
Corrected gestational age	691	525	103	63	0.163
	38.33 (2.78)	38.41 (2.92)	37.85 (2.42)	38.44 (1.84)	
Maternal age	708	525	106	77	0.082
	29.19 (5.50)	28.94 (5.61)	29.57 (5.11)	30.35 (5.17)	
ppBMI	544	428	62	54	0.007°
	27.98 (7.17)	27.55 (6.88)	28.55 (7.63)	30.72 (8.33)	

Variable	Total study population *n*(%)	Pre-COVID *n* = 525 (%)	During-COVID *n* = 107 (%)	Post-COVID *n* = 77 (%)	
Delivery mode					0.034*°
Vaginal	520 (73.4)	379 (72.2)	75 (70.8)	66 (85.7)	
C-section	188 (26.6)	146 (27.8)	31 (29.2)	11 (14.3)	
NICU					0.165
Yes	194 (27.4)	145 (27.6)	34 (32.1)	15 (19.5)	
No	514 (72.6)	380 (72.4)	72 (67.9)	62 (80.5)	
Race					0.297
White	458 (63.4)	333 (72.6)	77 (62.3)	48 (64.7)	
African American	102 (15.6)	82 (11.3)	12 (15.6)	12 (15.0)	
Hispanic	59 (8.8)	46 (6.6)	7 (13.0)	10 (8.9)	
Other/unknown	73 (12.2)	64 (9.4)	11 (9.1)	7 (11.4)	
Diabetes^a^					0.049^#^
Yes	94 (13.3)	60 (11.4)	20 (18.9)	14 (18.2)	
No	614 (86.7)	465 (88.6)	86 (81.1)	63 (81.8)	
Preeclampsia					0.226
Yes	73 (10.4)	50 (9.5)	16 (15.1)	7 (9.6)	
No	630 (89.6)	474 (90.5)	90 (84.9)	66 (90.4)	
COVID infection in pregnancy					0.087
Yes	17 (2.4)	0 (0.0)	6 (5.6)	11 (14.5)	
No	691 (97.6)	525 (100)	101 (94.4)	65 (85.5)	
COVID vaccination status					<0.001
Yes	76 (10.8)	0 (0.0)	24 (22.4)	52 (70.3)	
No	630 (89.2)	525 (100)	83 (77.6)	22 (29.7)	

^a^ Includes type I, type II, and gestational diabetes.

^*^ During- vs. Post-COVID.

^°^ Post- vs. pre-COVID significance.

^#^ Not significant after Tukey adjustment.

NICU, neonatal intensive care unit; ppBMI, pre-pregnancy body mass index; SD, standard deviation.

### Socioeconomic variables

Income was available for 404 mothers, and 276 (63.8%) mothers reported income >150% of the poverty level. Mothers in the during-COVID group were significantly more likely to report an income >150% of the poverty level compared with mothers in the pre-COVID group (*p* = 0.0385).

Food security questionnaires were available for 523 mothers, with 395 (75.5%) mothers reporting high food security, 54 (10.3%) mothers reporting marginal food security, and 74 (14.1%) mothers reporting low or very low food security. Mothers in the during-COVID group were significantly more likely to report high food security compared with mothers recruited pre-COVID (*p* < 0.0001). However, there was no significant between-group difference in self-reported barriers to accessing healthy foods, SNAP enrollment, or WIC enrollment.

For insurance type, 280 (40.2%) mothers had public insurance, 395 (56.7%) mothers had private insurance, and 22 (3.2%) mothers had self-pay insurance. There was a significant increase in mothers reporting private over public insurance when comparing pre-COVID with during-COVID (*p* < 0.001) and post-COVID (*p* = 0.04) groups.

Marital status was reported for 468 mothers, including 145 (31.0%) single mothers, 308 (65.8%) married mothers, and 15 (3.2%) divorced mothers. Women in the during-COVID (*p* = 0.04) and post-COVID (*p* < 0.001) groups were significantly more likely to be married compared with the pre-COVID group.

Information on housing was reported for 467 mothers, including 224 (48.0%) mothers who owned their home, 224 (48.0%) mothers who rented their home, and 19 (4.1%) mothers who had a different arrangement for housing. There was a significant increase in home ownership when comparing post- versus pre-COVID groups (*p* < 0.001). There were no significant differences in the other socioeconomic variables that we examined across the pandemic groups, including maternal education, employment status, or transportation. All variables are in Table 2.

**Table 2.** Socioeconomic Variables for Pre-, During-, and Post-COVID Groups

Variable	Pre-COVID-19 *N* = 525 (74.0%)	During COVID-19 *N* = 107 (15.1%)	Post-COVID-19 *N* = 77 (10.9%)	*p*-Value
Education				0.125
No high school diploma	17 (4.8%)	2 (3.4%)	0 (0.0%)	
High school	66 (18.6%)	7 (11.9%)	7 (13.5%)	
Some college	116 (32.7%)	16 (27.1%)	13 (25.0%)	
College graduate	156 (43.9%)	34 (57.6%)	32 (61.5%)	
Employment status				0.753
Full-time	206 (58.0%)	33 (54.1%)	27 (51.9%)	
Part-time	48 (13.5%)	10 (16.4%)	6 (11.5%)	
Unemployed	101 (28.5%)	18 (29.5%)	19 (36.5%)	
Income to poverty level				0.003^^^
>150% of poverty level	194 (63.8%)	45 (84.9%)	37 (78.7%)	
<150% of poverty level	110 (36.2%)	8 (15.1%)	10 (21.3%)	
Insurance type				<0.001^^^°
Public	232 (44.7%)	22 (21.6%)	26 (34.2%)	
Private	268 (51.6%)	77 (75.5%)	50 (65.8%)	
Self-pay	19 (3.7%)	3 (2.9%)	0 (0.0%)	
Marital status				
Single	128 (36.1%)	11 (18.0%)	6 (11.5%)	
Married	216 (60.8%)	47 (77.0%)	45 (86.5%)	0.001^^^°
Divorced	11 (3.1%)	3 (4.9%)	1 (1.9%)	
Rent vs. own home				
Own	156 (44.1%)	34 (55.7%)	34 (65.4%)	
Rent	179 (50.6%)	27 (44.3%)	18 (34.6%)	0.001°
Other	19 (5.4%)	0 (0.0%)	0 (0.0%)	
Transportation				
Own car	327 (92.9%)	58 (95.1%)	48 (92.3%)	
Shared car	15 (4.3%)	3 (4.9%)	3 (5.8%)	0.716
No car	10 (2.8%)	0 (0.0%)	1 (1.9%)	
Barriers to food				
Yes	16 (4.5%)	0 (0.0%)	2 (3.8%)	0.237
No	337 (95.5%)	61 (100.0%)	50 (96.2%)	
SNAP				
Yes	73 (21.0%)	10 (16.4%)	8 (15.4%)	0.499
No	275 (79.0%)	51 (83.6%)	44 (84.6%)	
WIC				0.028^#^
Yes	115 (33.0%)	12 (19.7%)	10 (19.6%)	
No	234 (67.0%)	49 (80.3%)	41 (80.4%)	
Food security category				0.010^^^
High	287 (71.9%)	54 (90.0%)	54 (84.4%)	
Marginal	47 (11.8%)	4 (6.7%)	3 (4.7%)	
Low	65 (16.3%)	2 (3.3%)	7 (10.9%)	

^^^ During- vs. pre-COVID significance.

^°^ Post- vs. pre-COVID significance.

^#^ Not significant after Tukey adjustment.

SNAP, Supplemental Nutrition Assistance Program; WIC, Women, Infants, and Children.

### Anxiety

Incidence of anxiety (*p* < 0.001), generalized anxiety disorder (*p* = 0.002), medication use for anxiety (*p* < 0.001), and history of anxiety (*p* < 0.001) were significantly higher in the during-COVID group compared with the pre-COVID group. Additionally, the incidence of anxiety (*p* < 0.001), medication for anxiety (*p* < 0.001), and history of anxiety (*p* < 0.001) were significantly higher in the post-COVID group compared with the pre-COVID group. All variables are in Table 3.

**Table 3.** Anxiety Variables During Pre-, During-, and Post-COVID Groups

Variable	Pre-COVID-19 *N* = 525 (74.0%)	During COVID-19 *N* = 107 (15.1%)	Post-COVID-19 *N* = 77 (10.9%)	*p*-Value
Anxiety				
Yes	101 (19.2%)	44 (41.1%)	41 (53.2%)	<0.001^^^°
No	424 (80.8%)	63 (58.9%)	36 (46.8%)	
GAD				0.002^^^
Yes	24 (4.6%)	14 (13.1%)	7 (9.7%)	
No	499 (95.4%)	93 (86.9%)	65 (90.3%)	
Anxiety medication use				<0.001^^^°
Yes	39 (7.4%)	26 (24.3%)	16 (21.3%)	
No	486 (92.6%)	81 (75.7%)	59 (78.7%)	
History of anxiety				<0.001^^^°
Yes	133 (25.3%)	45 (42.1%)	33 (42.9%)	
No	392 (74.7%)	62 (57.9%)	44 (57.1%)	

^^^ During- vs. pre-COVID significance.

^°^ Post- vs. pre-COVID significance.

GAD, generalized anxiety disorder.

### Depression

Incidence of depression (*p* < 0.001), major depressive disorder (MDD) (*p* < 0.001), medication use for depression (*p* < 0.001), history of depression (*p* = 0.004), and history of PPD (*p* < 0.001) were statistically higher in the during-COVID group compared with the pre-COVID group. Additionally, the incidence of MDD was significantly higher in the post-COVID group compared with the pre-COVID group. All variables are in Table 4.

**Table 4.** Depression Variables During Pre-, During-, and Post-COVID Groups

Variable	Pre-COVID-19 *n* = 525 (74.0%)	During COVID-19 *n* = 107 (15.1%)	Post-COVID-19 *n* = 77 (10.9%)	*p*-Value
Depression				<0.001^^^
Yes	92 (17.5%)	36 (33.6%)	18 (23.4%)	
No	433 (82.5%)	71 (66.4%)	59 (76.6%)	
MDD				<0.001^^^°
Yes	18 (3.4%)	11 (10.3%)	9 (12.2%)	
No	504 (96.6%)	96 (89.7%)	65 (87.8%)	
Depression medication use				<0.001^^^
Yes	52 (9.9%)	25 (23.4%)	13 (17.3%)	
No	473 (90.1%)	82 (76.6%)	62 (82.7%)	
History of depression				0.004^^^
Yes	140 (26.7%)	44 (41.1%)	29 (37.7%)	
No	385 (73.3%)	63 (58.9%)	48 (62.3%)	
History of PPD				<0.001^^^
Yes	26 (5.0%)	18 (16.8%)	6 (8.0%)	
No	495 (95.0%)	89 (83.2%)	69 (92.0%)	

^^^ During- vs. pre-COVID significance.

^°^ Post- vs. pre-COVID significance.

MDD, major depressive disorder; PPD, postpartum depression.

### Other psychiatric conditions

The incidence of other psychiatric conditions did not significantly differ between pandemic groups (12.0% pre-COVID vs. 14.0% during-COVID vs. 12.0% post-COVID; *p* = 0.849). However, medication use for other psychiatric conditions was significantly higher in the during-COVID (*p* < 0.001) and post-COVID (*p* < 0.001) groups compared with the pre-COVID group. Additionally, medication use for other mental health psychiatric conditions was significantly higher in the post-COVID group compared with the during-COVID group (*p* < 0.001). Out of 483 mothers with available data in the pre-COVID group, 7 (1.4%) mothers had medication use for other psychiatric conditions. Seven out of 107 (6.5%) mothers in the during-COVID group and 5 out of 16 (31.3%) mothers in the post-COVID group had medication use for other psychiatric conditions.

### Substance use

Incidence of substance use (*p* = 0.127), alcohol use (*p* = 0.577), and smoking status (*p* = 0.235) were not statistically significant across pandemic groups. All variables are in Table 5.

**Table 5.** Substance Use Variables for Pre-, During-, and Post-COVID Groups

Variable	Pre-COVID-19 *N* = 525 (74.0%)	During COVID-19 *N* = 107 (15.1%)	Post-COVID-19 *N* = 77 (10.9%)	*p*-Value
Substance use				0.127
Yes	46 (8.8%)	16 (15.0%)	6 (8.0%)	
No	477 (91.2%)	91 (85.0%)	69 (92.0%)	
Alcohol use				0.577
Yes	7 (1.3%)	1 (0.9%)	0 (0.0%)	
No	516 (98.7%)	105 (99.1%)	76 (100.0%)	
Smoking				0.235
None	389 (74.2%)	88 (84.6%)	59 (76.6%)	
Current	69 (13.2%)	9 (8.7%)	8 (10.4%)	
Former	66 (12.6%)	7 (6.7%)	10 (13.0%)	

## Discussion

Our study found significant increases in anxiety, depression, and medication use for other psychiatric conditions in the during-COVID compared with pre-COVID groups, and the incidence of these psychiatric conditions remained elevated in the post-COVID group. This concurs with previous studies that looked at the mental health of pregnant women during- versus pre-COVID. Increases in mental health conditions during the pandemic as compared with pre-pandemic levels were noted by studies such as Davenport et al., in which reports of anxiety increased from 29% to 72% and depression increased from 15% to 40.7% for a group of 900 women who were either pregnant or postpartum.^14^ Similarly, we observed a rising incidence of anxiety from 19.2% (101 mothers) in the pre-pandemic group to 41.1% (44 mothers) and 53.2% (41 mothers) in the during- and post-COVID groups, respectively; we also found a significant rise in the incidence of depression, with 17.5% (92 mothers) of women having a diagnosis of depression in the pre-COVID group compared with 33.6% (36 mothers) in the during-COVID group and 23.4% (18 mothers) in the post-COVID group. Interestingly, the incidence of anxiety continued to increase following the pandemic, while the incidence of depression decreased toward pre-pandemic levels. This may be due to the lifting of quarantining and lockdown measures, which could help to reduce social isolation and depression while simultaneously increasing anxiety due to concerns such as staying safe from COVID-19 and adjusting to in-person events.

Previous studies have focused on self-reported symptoms of anxiety and depression; in contrast, our determination of anxiety and depression relied on diagnoses of anxiety and depression in the medical record. It is possible that clinicians had an increased index of suspicion for mental health disorders such as anxiety and depression during the pandemic, due to the known social stressors associated with the pandemic. However, the pandemic also disrupted access to medical care, which may have resulted in reduced screening and treatment for women with mental health conditions.^22^ Our study finds similar increases in mental health conditions for pregnant women during- and post-COVID compared with other studies using self-reported symptoms of mental health conditions.^14^ These findings reinforce the notion that the mental health of pregnant women worsened during the COVID-19 pandemic and highlight the importance of ensuring adequate mental health screening and support during pregnancy.^7^

Environmental stressors also play an important role in determining the severity of mental health conditions experienced by pregnant women and the necessity for psychiatric medication use. During the pandemic, Moyer et al. highlighted that pregnant women in the United States experienced stressors from sources such as food insecurity, financial constraints related to income or employment status, and lack of in-home childcare—all of which can amplify the severity of mental health conditions.^23^ Several of these environmental stressors are tied to changes in the economic livelihood of women during the COVID-19 pandemic, especially women who worked in sectors such as hospitality and education, which experienced higher rates of job loss due to societal lockdowns.^24^

In our study, we found that women recruited during the height of the COVID-19 pandemic tended to have higher income and food security and were more likely to be married, own a home, and have private insurance compared with their pre-COVID peers. Federal programs implemented during the pandemic, such as stimulus payments, expansions to the SNAP, or expansions to Medicaid, may have impacted these results. Alternatively, this may be a result of our study exclusively recruiting COVID-negative patients during COVID due to hospital safety policies. It is possible that women who tested negative for COVID-19 had access to more economic resources, which may have protected them from COVID infection and may explain the observed differences in maternal socioeconomic status between the pre- and during-COVID groups. In addition, these findings may have also arisen due to mistrust of research by mothers from historically disadvantaged groups that prevented them from participating in our study. However, although women who were recruited into the during-COVID group had greater access to resources than women in the pre-COVID group, women in the during-COVID group were still more likely to experience changes in their mental health compared with their pre-COVID peers. This indicates that women during COVID experienced various combinations of stressors, regardless of their economic situations. Many pregnant women suddenly became full-time care providers for their children and family while managing employment responsibilities (either remote or in-person) and recovering from pregnancy, sometimes with limited support from other family members and friends.^25^ In sum, the COVID-19 pandemic exposed pregnant women to extreme familial, occupational, social, and financial strains and uncertainty, all of which culminated in increased vulnerability to developing mental health conditions. Knowing that mental health in pregnant women worsened during the pandemic, it is especially important to identify environmental stressors that exacerbated these shifts, with the ultimate aim of reducing or eliminating these stressors.

With a growing body of research demonstrating a significant increase in maternal mental health conditions during the height of the COVID-19 pandemic, it is paramount that clinicians implement guidelines for early mental health assessment and intervention in the case of future epidemics and pandemics.^23,25,26^ Dennis and Chung-Lee indicated that therapies for pregnant women may range from psychiatric medications to therapy visits, peer and family support, and exercise, indicating the range of treatments that can help support pregnant women.^27^ While many of the women in our study were prescribed talk therapy or psychiatric medications for treatment, therapies such as peer and family support and exercise were not as commonly discussed with patients. This may result partially from the effects of social isolation and quarantining, which limit the range of non-medication therapies that physicians have at hand during pandemics and lockdowns.

Limitations in our study included differences in enrollment numbers across the three periods due to restrictions on enrollment in the during- and post-COVID periods at our academic medical center. This may have impacted our study’s ability to adequately capture a diverse sample population in the during- and post-COVID periods. Additionally, appointment limitations and staffing shortages impacted the rates of Edinburgh Postnatal Depression Scale (EPDS) screening in during- and post-COVID. This led to low rates of EPDS scores in the EMR and affected our study’s ability to use EPDS, an excellent measure for PPD. As a result, we were unable to assess differences in PPD for the current pregnancy, but we did identify differences in the history of PPD across the pandemic time points. Finally, this study only enrolled pregnant women, so it is not possible to evaluate the impact of the COVID-19 pandemic in pregnant women compared with nonpregnant women in the general population. However, previous research has shown that pregnant women have unique risk factors for developing anxiety and depression,^4,5^ and our study underscores the high prevalence of maternal mental health conditions in the Midwest United States, particularly during the COVID-19 pandemic. More research is needed to determine appropriate, holistic interventions to support maternal mental health recovery following a pandemic, particularly for mothers impacted by environmental stressors such as financial strain, which may have been exacerbated by the pandemic.

## Conclusions

This study is one of the first to examine maternal mental health, medication use, substance use, and socioeconomic variables across pre-, during-, and post-COVID time points. When evaluating these groups, we observed a pandemic-associated increased risk for the development of mental health disorders and psychiatric medication use among pregnant women. Previous studies have shown that pregnant women are at risk of experiencing anxiety, depression, and other mental health conditions during pregnancy. Our study supports these findings and indicates an increase in these conditions in the setting of and following the pandemic. Research identifying specific strategies to mitigate the development of perinatal mental health disorders during a pandemic is necessary to drive the development of support systems for maternal mental health during special times such as epidemics and pandemics.

## Data Sharing Statement

Due to the confidentiality of subject data, data is available upon request to the corresponding author.

## Abbreviations Used

ANOVAs: Analyses of Variance
C-section: cesarean section
CGA: Corrected Gestational Age
COVID-19: coronavirus disease 2019
EHR: Electronic Health Record
EPDS: Edinburgh Postnatal Depression Scale
GAD: Generalized anxiety disorder
IRB: Institutional Review Board
MDD: Major Depressive disorder
PPD: Post Partum Depression
SARS-CoV-2: Severe acute respiratory syndrome coronavirus 2
SDs: Standard Deviations
SNAP: Supplemental Nutritional Assistance Program
U.S.: United States
WIC: Women Infants and Children
